# Knowledge, Attitude, and Practice (KAP) of Radiographic Protection by Dental Undergraduate and Endodontic Postgraduate Students, General Practitioners, and Endodontists

**DOI:** 10.1155/2020/2728949

**Published:** 2020-04-27

**Authors:** Amal A. Almohaimede, Mohammad W. Bendahmash, Feras M. Dhafr, Abdullah F. Awwad, Ebtissam M. Al-Madi

**Affiliations:** ^1^Department of Restorative Dental Sciences, Division of Endodontics, College of Dentistry, King Saud University, P.O. Box 5967, Riyadh 11432, Saudi Arabia; ^2^Dental College, King Saud University, P.O. Box 5967, Riyadh 11432, Saudi Arabia; ^3^College of Dentistry, Riyadh Elm University, Alnamuthajiyah Campus, Riyadh 12734, Saudi Arabia

## Abstract

The aim of this study is to evaluate the knowledge, attitude, and perception of radiation hazard and preventive measures among dental undergraduate students, general practitioners, endodontic postgraduate students, and endodontists in Saudi Arabia. Multiple choice questions questionnaires were distributed among undergraduate and endodontic postgraduate dental students, general practitioners, and endodontists in the colleges of dentistry in Saudi Arabia, government hospitals, and private clinics. The questionnaire included sociodemographic data, assessment of the knowledge of radiation physics and biology, assessment of the practice of dental radiography, and assessment of knowledge of radiation protection. Chi-square test was used for individual and multiresponse analysis. Level of statistical significance was set at *P* ≤ 0.05. Three hundred and twenty-nine responded to the questionnaire. More than half of the respondents agreed that dental X-ray is hazardous to health (60.79%), and 68.1% were familiar with ALARA (as low as reasonably achievable) principle. However, only 34% are familiar with the recommendations of the National Council on Radiation Protection (NCRP) and International Commission on Radiological Protection (ICRP). The use of lead apron and thyroid collar for patients' protection from X-ray radiation was practiced among endodontic postgraduate students more frequently as well as those who are proactive in the academic field. Undergraduate students, endodontic postgraduate students, and endodontists in the academic field were the most aware towards radiation reduction measures. The use of the preventive measures needs to be emphasized more among general practitioners, endodontic postgraduate students, and endodontists especially in governmental hospitals and private sectors.

## 1. Introduction

Since the introduction of the X-ray and its uses in dental radiology in 1895, it has been a very important diagnostic method, especially in modern dentistry [1]. The ability of the ionizing radiation to penetrate the soft tissue to reflect an image that cannot be seen by the human eye on a sensor gives it a great importance in several branches of dentistry. Its usage varies from diagnosing minor caries lesions to diagnosing periapical and maxillofacial lesions. Nevertheless, ionizing radiation could be biologically damaging to living tissues [2]. It may directly damage the DNA of the living cell and indirectly by creating free radicals, which are unstable and reactive uncharged molecules. Unstable radicals tend to stabilize by rebinding, and this could form new toxic substances, such as hydrogen peroxide [H_2_O_2_], which can lead to cellular alternations [3]. It has been postulated that repeated exposure to cytotoxic materials can result in chronic cell injury, compensatory cell proliferation, hyperplasia, and, ultimately, tumor development [2]. In endodontics, several radiographic images are required during treatment. Therefore, there is an increase in the dosage received by the patient, which in turn means there is a greater risk for the patient in addition to the healthcare provider, if precautions are not followed.

The effects of the X-ray on the living tissue are deterministic and stochastic. Both are harmful and could lead to serious complications [4]; therefore, it is very important to understand the potential risks and how to apply the precautions and preventive measures correctly. Previous studies done in the Kingdom of Saudi Arabia found that knowledge, attitude, and practice level with regard to radiation protection was higher among dental students, and the least was among dental staff [5, 6].

We hypothesize that the knowledge and the perception of dental undergraduate students, general practitioners, endodontic postgraduate students, and endodontists on radiographic protection is high, but the attitude towards taking precautions might be low.

The aim of this study is to evaluate the knowledge, attitude, and perception of radiation hazard and preventive measures of the ionizing radiation among dental undergraduate students, general practitioners, endodontic postgraduate students, and endodontists in Saudi Arabia.

## 2. Subjects and Methods

The present study was approved by the Institutional Review Board of Princess Noura Bint Abdulrahman University (H-01-R-059). Dental students and clinical practitioners that deal with radiography during their clinical practice were sent questionnaires related to knowledge, attitude, and perception of radiation hazard and preventive measures of ionizing radiation. The questionnaire was distributed among undergraduate and postgraduate endodontic dental students, staff, and faculty (general practitioners and endodontists) in the colleges of dentistry at King Saud University (KSU), Princess Noura Bint Abdulrahman University (PNU), King Saud Bin Abdulaziz University for Health Sciences (KSAUHS), King Abdulaziz University (KAU), King Faisal University (KFU), Imam Abdulrahman Bin Faisal University (IAU), Umm Alqura University (UQU), King Khalid University (KKU), Taiba University (TU), Qassim University (QU), Prince Sattam University (PSAU), Hail University (UOH), Almajmaa University (MU), Riyadh Elm University (REU), and Alfarabi Colleges. Moreover, it was sent to clinicians (general practitioners and endodontists) in government hospitals including Ministry of Health Dental Clinics (MOH), Security Forces Hospital (SFH), King Abdulaziz Medical City (KAMC), Prince Sultan Medical Military City (PSMMC), and private practice.

The form was electronic based and distributed online through Google Forms by Twitter and WhatsApp social media platforms.

The questionnaire was in the form of multiple choice questions related to the harmful ionizing radiation and protective measures. It was developed in English and a pilot test of 25 questionnaires was performed against a checklist to determine content clarity, language development, and validity. The questionnaire was divided into three sections: sociodemographic data that consisted of gender, age range, working place, years of clinical experience, and socioeconomic status. Assessment of the knowledge of radiation physics and biology, assessment of the practice of dental radiography, and assessment of knowledge of radiation protection were asked in the questionnaire.

## 3. Statistical Analysis

Data were entered into an electronic database and analyzed using SPSS (Statistical Package for the Social Sciences) version 20 (SPSS Inc., Chicago, IL, USA). Descriptive statistics were used. For differential statistics, the chi-square test was used for individual and multiresponse analysis. Level of statistical significance was set at *P* ≤ 0.05.

## 4. Results

Demographic data results are summarized in Table 1. Three hundred and twenty-nine respondents (329) completed questionnaires. More than half of the respondents agreed that dental X-ray is hazardous to health (60.79%) and can cause DNA alteration (59.87%). Most of the respondents (70.8%) mentioned that the damage caused by X-ray is mainly due to the formation of free radicals. In this statement, there was statistical significance between the different educational levels (*P* < 0.05), as undergraduate students showed most of the correct answers. On the other hand, most of the respondents (81.5%) agreed that radiation from dental X-ray accumulates over time, while 64.4% agreed that damage to living tissues by radiation is due to direct and indirect effect, and 61.1% agreed that short-term effects of radiation are due to large amount of radiation absorbed over a short period of time. Only 42.6% agreed that the cause of long-term effects of radiation is the small amount of radiation absorbed repeatedly over a long period of time. Almost half the respondents (53.2%) agreed that X-rays can be reflected from the walls of the room. In these responses, there was no statistically significant difference between the different educational levels (*P* > 0.05).

Most of the respondents use radiographs frequently in different stages of their endodontic treatment, either preoperatively for case evaluation (83%), or during treatment for verification of the procedure (78.1%), or postoperatively to evaluate treatment outcome (76%).

Tables 2 and 3 show the practitioners' awareness and knowledge towards radiation protection according to their educational level and working area. Endodontists and endodontic postgraduate students showed better knowledge in some questions compared to the undergraduate students and general practitioners as specified in Table 2. Regarding the working area, most of the respondents had the same level of knowledge except in question 1 (familiarity with the ALARA principle), where the academic and the government sectors showed better results than the private sector (Table 3). Almost 68.1% of the respondents mentioned that they are familiar with ALARA (as low as reasonably achievable) principle. On the other hand, only 34% are familiar with the recommendations of the National Council on Radiation Protection (NCRP) and International Commission on Radiological Protection (ICRP). Most respondents (84.5%) are aware of the radiation hazard symbol. The importance of the use of collimators and filters in dental radiography was approved by 88.4% of the respondents. Furthermore, 79.9% of the respondents agreed that digital radiography requires less exposure than the conventional radiography. Moreover, 73.6% of the participants agreed that high-speed films reduce the exposure. Most respondents (72.3%) agreed that ideal distance of operator (Position Distance Rule) when exposed to dental radiation is 6 ft, 90°–135°. Tables 4 and 5 show practitioners' attitude and practice towards radiation protection according to their educational level and working area. Results showed that the undergraduate students were the best in following the radiation protection measures, and the least were the endodontists as specified in Table 4. Academic sector showed better results in following the radiation protection measures compared to the government sector and private practice as specified in Table 5.

## 5. Discussion

Successful endodontic treatment requires adjunctive tools, such as intraoral radiographs, to be used preoperatively during endodontic examination and case evaluation, or during treatment for verification of the procedure, or postoperatively to evaluate treatment outcome. In this study, the frequency of taking radiographs during dental procedure differentiate statistically between the different educational groups (*P* < 0.05), where the endodontic postgraduate students and endodontists were mostly taking three and more radiographs compared to less number of radiographs taken by undergraduate students and general practitioners. These results were in accordance with previous studies, where they found that the number of radiographs taken during root canal treatment varies between three to four radiographs [7, 8]. This can be explained by the type of the dental procedure that the practitioner is doing, where two radiographs are minimally needed while performing root canal treatment (preoperative and postoperative radiographs) to adhere to the acceptable standard of care [9, 10].

The results showed that the awareness of radiation danger among dental practitioners was not completely sufficient. This finding was in agreement with a previous study from the Medical University of Warsaw [11]. Undergraduate students performed better at choosing the correct answers. This was in contrast to a previous study that assessed the knowledge of Saudi undergraduate dental students towards the risks of dental X-ray [6] and a study that showed that specialist had higher level of knowledge [11–13]. This could be explained by the current structured radiological courses that are included in their curriculum.

Despite the frequent use of radiographs by the respondents, the results showed that they do not sufficiently follow the American Dental Association guidelines for protection of patients and practitioners. The users of the thyroid collar were 66.9% and 80.2% for the lead apron. The least users were general practitioners and practitioners in private practice. This was in agreement with previous studies in Korea, Belgium, and India [13–16]. In this study, most users of lead aprons and thyroid collar were dental undergraduate students, which was in agreement with a previous study done on North American dental schools [17].

A previous study evaluated the shielding effect of the thyroid collar for digital panoramic radiography and showed that wearing the thyroid collar was helpful when direct digital panoramic imaging systems were used [18]. Moreover, Hoogeveen et al. concluded that the thyroid shield helps in reduction of the dose to the thyroid when imaging the upper anterior teeth [19]. Schueler showed that 0.5 mm thickness apron constricted 90% of the scatter radiation [20]. On the other hand, Hyun et al. quantified the level of 0.5 mm thick lead apron in blocking radiation, and they found that it blocked just over one third of it [21]. The American Dental Association recommendations for patient shielding are to use protective thyroid collars whenever possible, while the use of abdominal shielding may not be necessary [22]. The harmful effects of dental X-rays on the thyroid gland have been proven by several studies, where they concluded that repeated exposures to dental X-rays might be associated with an increased risk of thyroid cancer [23, 24]. Moreover, brain tumors, tumors of head and neck areas, and harmful health outcomes resulting from exposure to dental diagnosis X-rays have been reported [25]. One case report documented the harmful effect of holding the film in patient's mouth during exposure [26].

To reduce the occupational dose, which is defined by the International Commission of Radiological Protection as “the exposure incurred at work and principally as a result of work,” three factors should be considered: the shielding of walls, the position of the X-ray beam that should be directed towards a shielded area, and the distance of the operator when exposed to dental radiation that should be 6 feet (2 meters) away from the patient at an angle of 90°–135° from the tube head (Position Distance Rule) [27]. In 2014, at the National Council on Radiation Protection and Measurements (NCRP) Annual Meeting, Bushberg introduced the term ALADA (as low as diagnostically acceptable) as a variation of the acronym ALARA (as low as reasonably achievable) to stress the value of optimization in medical imaging, which means that the radiograph should be of acceptable diagnostic quality, with the minimum dose to the patient [28]. Furthermore, the use of digital sensors or F-speed film (the fastest among other types) along with rectangular collimation should be considered to minimize radiation exposure [29]. More mesaures to reduce the radiation exposure is the use of receptor holders to optimize and minimize repeated exposure [30].

This study had some limitations where the number of respondents could not be controlled. Moreover, the number of respondents varied among specialities and working sectors and not equally distributed.

## 6. Conclusion

Within the limitations of the study, it can be concluded that the knowledge of radiation hazard and preventive measures of the ionizing radiation among dental undergraduate students, endodontic postgraduate students, and endodontists in Saudi Arabia was quite fair especially in academic field. However, the radiation protection measures need to be emphasized more among general practitioners, endodontic postgraduate students, and endodontists especially in governmental hospitals and private sectors.

## Figures and Tables

**Table 1:** Sociodemographic data.

Sociodemographic data	Percentage (%)
Gender	
Male	38.9
Female	61.09
Age	
20-25	40.7
26-30	22.2
31-35	12.5
36-40	13.7
41-45	4.3
>45	6.7
Educational level	
Undergraduate students	35
Endodontic postgraduate students	20.7
General practitioners	23.1
Endodontists	21
Years of clinical experience	
1–5 years	54.1
6–10 years	16.1
11–15 years	7.9
>15 years	10.6
Working place	
Academic sector	73.11
KSU	30.49
KSAUHS	4.91
PNU	6.88
KAU	3.27
KFU	0.65
IAU	1.63
UQU	0.32
KKU	3.27
TU	0.32
QU	0.65
PSAU	0.65
UOH	0.65
MU	0.98
REU	18.03
Alfarabi colleges	0.32
Government sector	23.6
MOH	17.7
SFH	0.98
KAMC	1.63
PSMMC	3.27
Private sector	3.27

**Table 2:** Practitioners' awareness and knowledge towards radiation protection according to their educational level.

No.	Knowledge items	Response	Educational level				*P*-value
			Undergraduate students (%)	Endodontic postgraduate students (%)	General practitioners (%)	Endodontists (%)	
1	Are you familiar with ALARA principle?	**Yes**	74.8^a^	75^a^	48.7^b^	72.5^a^	**0.001** ^*∗*^
		**No**	25.2	25	51.3	27.5	

2	Are you familiar with the recommendations of the NCRP and ICRP?	**Yes**	29.6	38.2	28.9	43.5	**0.16**
		**No**	70.4	61.8	71.1	56.5	

3	Are you aware of the radiation hazard symbol?	**Yes**	80.9	83.8	82.9	92.8	**0.179**
		**No**	19.1	16.2	17.1	7.2	

4	Does digital radiography require less exposure than conventional?	**Yes**	72.2^a^	85.3^b^	75^a^	94.2^b^	**0.014** ^*∗*^
		**No**	14.8	8.8	11.8	2.9	
		**I do not know**	13	5.9	13.2	2.9	

5	Do high-speed films reduce exposure?	**Yes**	65.2^a^	77.9^b^	69.7^a^	88.4^b^	**0.002** ^*∗*^
		**No**	9.6	8.8	2.6	7.2	
		**I do not know**	25.2	13.2	27.6	4.3	

6	Specify the importance of the use of collimators and filters in dental radiography.	**Very important** **+** **important**	85.2	94.1	81.6	95.7	**0.144**
		**Moderately important**	8.7	2.9	10.5	4.3	
		**Of little importance** **+** **unimportant**	6	2.9	7.9	0	

7	What is the ideal distance of operator “Position Distance Rule” when exposed to dental radiography?	**4ft., 90 ** ^**o**^ ** -135 ** ^**o**^	7.8	1.5	5.3	1.4	**0.084**
		**4ft, 60 ** ^**o**^ ** -90 ** ^**o**^	9.6	4.4	10.5	5.8	
		**6ft, 90 ** ^**o**^ ** -135 ** ^**o**^	67	86.8	64.5	76.8	
		**6ft, 60 ** ^**o**^ ** -90 ** ^**o**^	15.7	7.4	19.7	15.9	

^*∗*^Significant at *P* < 0.05. The same superscript letters indicate no significant differences (*P* > 0.05).

**Table 3:** Practitioners' awareness and knowledge towards radiation protection according to their working area.

No.	Knowledge items	Response	Working area			*P*-value
			Academic sector (%)	Government sector (%)	Private sector (%)	
1	Are you familiar with ALARA principle?	**Yes**	72.2^a^	61.1^a^	30^b^	**0.007** ^*∗*^
		**No**	27.8	38.9	70	

2	Are you familiar with the recommendations of the NCRP and ICRP?	**Yes**	33.6	36.1	30	**0.893**
		**No**	66.4	63.9	70	

3	Are you aware of the radiation hazard symbol?	**Yes**	83.4	84.7	100	**0.368**
		**No**	16.6	15.3	0	

4	Does digital radiography require less exposure than conventional?	**Yes**	76.2	91.7	70	**0.056**
		**No**	12.6	4.2	10	
		**I do not know**	11.2	4.2	20	

5	Do high-speed films reduce exposure?	**Yes**	70.4	81.9	80	**0.334**
		**No**	8.5	5.6	0	
		**I do not know**	21.1	12.5	20	

6	Specify the importance of the use of collimators and filters in dental radiography.	**Very important** **+** **important**	87.4	88.9	80	**0.1**
		**Moderately important**	6.7	9.7	10	
		**Of little importance** **+** **unimportant**	5.8	1.4	10	

7	What is the ideal distance of operator “Position Distance Rule” when exposed to dental radiography?	**4ft., 90 ** ^**o**^ ** -135 ** ^**o**^	5.8	4.2	0	**0.656**
		**4ft, 60 ** ^**o**^ ** -90 ** ^**o**^	8.5	5.6	20	
		**6ft, 90 ** ^**o**^ ** -135 ** ^**o**^	71.7	72.2	60	
		**6ft, 60 ** ^**o**^ ** -90 ** ^**o**^	13.9	18.1	20	

^*∗*^Significant at *P* < 0.05. The same superscript letters indicate no significant differences (*P* > 0.05).

**Table 4:** Practitioners' attitude and practice towards radiation protection according to their educational level.

No.	Attitude items	Response	Educational level				*P*-value
			Undergraduate students (%)	Endodontic postgraduate students (%)	General practitioners (%)	Endodontists (%)	
1	Do you use lead apron for patients during exposure?	**Very frequently** **+** **frequently**	85.2^a^	88.2^a^	75^b^	69.6^b^	**0.017** ^*∗*^
		**Occasionally**	9.6	4.4	11.8	11.6	
		**Rarely** **+** **never**	5.2	7.3	13.1	18.8	

2	Do you use thyroid collar for patients during exposure?	**Very frequently** **+** **frequently**	75.7^a^	76.5^a^	55.2^b^	55^b^	**0.001** ^*∗*^
		**Occasionally**	10.4	7.4	15.8	14.5	
		**Rarely** **+** **never**	13.9	16.2	28.9	30.4	

3	Do you ask patients to hold the film?	**Very frequently** **+** **frequently**	42.6^a^	54.4^b^	47.4^a^	56.5^b^	**0.019** ^*∗*^
		**Occasionally**	21.7	14.7	23.7	20.3	
		**Rarely** **+** **never**	35.7	30.9	29	23.2	

4	Do you stand directly in the path of the primary radiation?	**Very frequently** **+** **frequently**	10.4	10.3	10.6	11.6	**0.089**
		**Occasionally**	13.9	19.1	17.1	15.9	
		**Rarely** **+** **never**	75.7	70.6	72.4	72.4	

5	Do you stand behind a lead barrier during exposure?	**Very frequently** **+** **frequently**	66.1^a^	52.9^b^	55.3^b^	34.8^c^	**0.001** ^*∗*^
		**Occasionally**	18.3	23.5	17.1	14.5	
		**Rarely** **+** **never**	15.6	23.6	27.6	50.7	

6	If within the same area, do you stand 6 feet away from primary X-ray beam during exposure?	**Very frequently** **+** **frequently**	54.8	66.2	44.7	71	**0.053**
		**Occasionally**	21.7	20.6	30.3	14.5	
		**Rarely** **+** **never**	23.4	13.2	25	14.4	

7	Do you hold the film in the patients' mouth during exposure?	**Very frequently** **+** **frequently**	7.8^a^	19.1^b^	21^b^	15.9^b^	**0.001** ^*∗*^
		**Occasionally**	15.7	19.1	11.8	17.4	
		**Rarely** **+** **never**	76.5	61.8	67.1	66.7	

8	Do you stay within the same clinic during X-ray exposure?	**Very frequently** **+** **frequently**	16.5	23.6	21.1	26	**0.33**
		**Occasionally**	24.3	23.5	13.2	13	
		**Rarely** **+** **never**	59.2	53	65.7	60.9	

9	If you decided to stay within the same clinic during X-ray exposure, do you use lead apron on a regular basis?	**Very frequently** **+** **frequently**	41.7^a^	25^b^	32.9^b^	26.1^b^	**0.041** ^*∗*^
		**Occasionally**	13.9	19.1	13.2	10.1	
		**Rarely** **+** **never**	44.4^a^	55.8^d^	53.9^d^	63.7^d^	

10	Do you display caution or hold a warning sign while exposed to X-ray?	**Very frequently** **+** **frequently**	44.4^a^	33.8^b^	42.1^a^	26^b^	**0.03** ^*∗*^
		**Occasionally**	14.8	20.6	15.8	17.4	
		**Rarely** **+** **never**	40.9	45.5	42.1	56.5	

11	Do you allow people to come inside the room during exposure to X-ray?	**Very frequently** **+** **frequently**	3.4	3	5.3	0	**0.454**
		**Occasionally**	10.4	8.8	9.2	10.1	
		**Rarely** **+** **never**	86.1	88.2	85.6	89.9	

^*∗*^Significant at *P* < 0.05. The same superscript letters indicate no significant differences (*P* > 0.05).

**Table 5:** Practitioners' attitude and practice towards radiation protection according to their working area.

No.	Attitude items	Response	Educational level			*P*-value
			Academic sector (%)	Government sector (%)	Private sector (%)	
1	Do you use lead apron for patients during exposure?	**Very frequently** **+** **frequently**	86.6^a^	68.1^b^	50^c^	**0.001** ^*∗*^
		**Occasionally**	7.6	12.5	10	
		**Rarely** **+** **never**	5.8	19.4	40	

2	Do you use thyroid collar for patients during exposure?	**Very frequently** **+** **frequently**	73.6^a^	52.8^b^	30^c^	**0.004** ^*∗*^
		**Occasionally**	10.3	13.9	20	
		**Rarely** **+** **never**	16.1	33.3	50	

3	Do you ask patients to hold the film?	**Very frequently** **+** **frequently**	44.9^a^	65.3^b^	50^a^	**0.014** ^*∗*^
		**Occasionally**	21.1	18.1	30	
		**Rarely** **+** **never**	34	16.7	20	

4	Do you stand directly in the path of the primary radiation?	**Very frequently** **+** **frequently**	10.8	8.4	10	**0.623**
		**Occasionally**	15.2	13.9	20	
		**Rarely** **+** **never**	74	77.8	70	

5	Do you stand behind a lead barrier during exposure?	**Very frequently** **+** **frequently**	60.1^a^	38.9^b^	40^b^	**0.003** ^*∗*^
		**Occasionally**	18.8	18.1	0	
		**Rarely** **+** **never**	21.1	43.1	60	

6	If within the same area, do you stand 6 feet away from primary X-ray beam during exposure?	**Very frequently** **+** **frequently**	57.4	62.5	50	**0.641**
		**Occasionally**	21.1	22.2	10	
		**Rarely** **+** **never**	21.6	15.3	40	

7	Do you hold the film in the patients' mouth during exposure?	**Very frequently** **+** **frequently**	14.4^a^	11.1^a^	30^b^	**0.021** ^*∗*^
		**Occasionally**	13.9	20.8	10	
		**Rarely** **+** **never**	71.8	68	60	

8	Do you stay within the same clinic during X-ray exposure?	**Very frequently** **+** **frequently**	21.1	19.5	30	**0.613**
		**Occasionally**	20.2	16.7	10	
		**Rarely** **+** **never**	58.7	63.8	60	

9	If you decided to stay within the same clinic during X-ray exposure, do you use lead apron on a regular basis?	**Very frequently** **+** **frequently**	36.8^a^	27.8^a^	0^b^	**0.009** ^*∗*^
		**Occasionally**	14.8	6.9	10	
		**Rarely** **+** **never**	48.4	65.3	90	

10	Do you display caution or hold a warning sign while exposed to X-ray?	**Very frequently** **+** **frequently**	40.4	27.8	50	**0.352**
		**Occasionally**	17	15.3	10	
		**Rarely** **+** **never**	42.6	57	40	

11	Do you allow people to come inside the room during exposure to X-ray?	**Very frequently** **+** **frequently**	3.1	1.4	10	**0.534**
		**Occasionally**	10.8	6.9	10	
		**Rarely** **+** **never**	86.1	91.7	80	

^*∗*^Significant at *P* < 0.05. The same superscript letters indicate no significant differences (*P* > 0.05).

## Data Availability

The data used to support the findings of this study were supplied by the corresponding author under license and so cannot be made freely available. Requests for access to these data should be made to Amal Almohaimede (aalmohaimede@ksu.edu.sa).

## References

[1] Jones C. G. (2005). A Review of the history of U.S. radiation protection regulations, recommendations, and standards. *Health Physics*.

[2] Ribeiro D. A., de Oliveira G., de Castro G., Angelieri F. (2008). Cytogenetic biomonitoring in patients exposed to dental X-rays: comparison between adults and children. *Dentomaxillofacial Radiology*.

[3] Iannucci J. M., Howerton L. J. (2012). *Dental Radiography: Principles and Techniques*.

[4] Briggs-Kamara M., Okoye P., Omubo-Pepple V. B. (2013). Radiation safety awareness among patients and radiographers in three hospitals in Port Harcourt. *American Journal of Scientific and Industrial Research*.

[5] Basheer B., Albawardi K., Alsanie S. (2019). Knowledge, attitudes and perception toward radiation hazards and protection among dental professionals in Riyadh, Kingdom of Saudi Arabia. *International Journal of Medical Research & Health Sciences*.

[6] Arnout E. A., Jafar A. (2014). Awareness of biological hazards and radiation protection techniques of dental imaging- a questionnaire based cross-sectional study among Saudi dental students. *Journal of Dental Health, Oral Disorders & Therapy*.

[7] Chandler N. P., Koshy S. (2002). Radiographic practices of dentists undertaking endodontics in New Zealand. *Dentomaxillofacial Radiology*.

[8] Palmer N. O. A., Ahmed M., Grieveson B. (2009). An investigation of current endodontic practice and training needs in primary care in the north west of England. *British Dental Journal*.

[9] Simon S., Machtou P., Adams N., Tomson P., Lumley P. (2009). Apical limit and working length in endodontics. *Dental Update*.

[10] Raoof M., Heidaripour M., Shahravan A. (2014). General dental practitioners’ concept towards using radiography and apex-locators in endodontics. *Iranian Endodontic Journal*.

[11] Furmaniak K. Z., Kołodziejska M. A., Szopiński K. T. (2016). Radiation awareness among dentists, radiographers and students. *Dentomaxillofacial Radiology*.

[12] Svenson B., Söderfeldt B., Gröndahl H. (1997). Knowledge of oral radiology among Swedish dentists. *Dentomaxillofacial Radiology*.

[13] Aravind B. S., Joy E. T., Kiran M. S., Sherubin J. E., Sajesh S., Manchil P. R. D. (2016). Attitude and awareness of general dental practitioners toward radiation hazards and safety. *Journal of Pharmacy and Bioallied Sciences*.

[14] Lee B.-D., Ludlow J. B. (2013). Attitude of the Korean dentists towards radiation safety and selection criteria. *Imaging Science in Dentistry*.

[15] An S.-Y., Lee K.-M., Lee J.-S. (2018). Korean dentists’ perceptions and attitudes regarding radiation safety and protection. *Dentomaxillofacial Radiology*.

[16] Jacobs R., Vanderstappen M., Bogaerts R., Gijbels F. (2004). Attitude of the Belgian dentist population towards radiation protection. *Dentomaxillofacial Radiology*.

[17] Geist J. R., Katz J. O. (2002). Radiation dose-reduction techniques in North American dental schools. *Oral Surgery, Oral Medicine, Oral Pathology, Oral Radiology, and Endodontology*.

[18] Han G.-S., Cheng J.-G., Li G., Ma X.-C. (2013). Shielding effect of thyroid collar for digital panoramic radiography. *Dentomaxillofacial Radiology*.

[19] Hoogeveen R. C., Hazenoot B., Sanderink G. C. H., Berkhout W. E. R. (2016). The value of thyroid shielding in intraoral radiography. *Dentomaxillofacial Radiology*.

[20] Schueler B. A. (2010). Operator shielding: how and why. *Techniques in Vascular and Interventional Radiology*.

[21] Hyun S. J., Kim K. J., Jahng T. A., Kim H. J. (2016). Efficiency of lead aprons in blocking radiation-how protective are they?. *Heliyon*.

[22] American Dental Association Council on Scientific Affairs (2006). The use of dental radiographs: update and recommendations. *The Journal of the American Dental Association*.

[23] Memon A., Godward S., Williams D., Siddique I., Al-Saleh K. (2010). Dental x-rays and the risk of thyroid cancer: a case-control study. *Acta Oncologica*.

[24] Neta G., Rajaraman P., Berrington de Gonzalez A. (2013). A prospective study of medical diagnostic radiography and risk of thyroid cancer. *American Journal of Epidemiology*.

[25] Hwang S. Y., Choi E. S., Kim Y. S., Gim B. E., Ha M., Kim H. Y. (2018). Health effects from exposure to dental diagnostic X-ray. *Environmental Health and Toxicology*.

[26] Halboub E. S., Barngkgei I., Alsabbagh O., Hamadah O. (2015). Radiation-induced thumbs carcinoma due to practicing dental X-ray. *Contemporary Clinical Dentistry*.

[27] Crane G., Abbott P. (2016). Radiation shielding in dentistry: an update. *Australian Dental Journal*.

[28] Berkhout W. E. R. (2015). Het ALARA-principe. Achtergronden en toepassing in de praktijk. *Nederlands Tijdschrift Voor Tandheelkunde*.

[29] Okano T., Sur J. (2010). Radiation dose and protection in dentistry. *Japanese Dental Science Review*.

[30] Senior A., Wenand C., Ganatra S., Lai H., Alsulfyani N., Pachêco-Pereira C. (2018). Digital intraoral imaging re-exposure rates of dental students. *Journal of Dental Education*.

